# Supplementary material for: Self-reported depression and anxiety rates among females with cutaneous leishmaniasis in Hubuna, Saudi Arabia
Source: PeerJ. 2023 Jun 20;11:e15582. doi: 10.7717/peerj.15582 (PMC10289083; doi:10.7717/peerj.15582)
Supplement: Supplemental Information 2 [file peerj-11-15582-s002.docx]

**
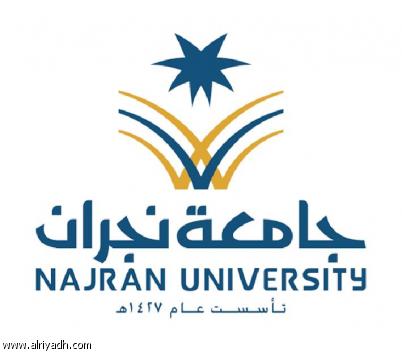
كلية التمريض**

============================================================

Study questionnaire

Women's perception of cutaneous leishmaniasis: Anxiety and depression at Habouna area- Saudi Arabia

**To the participants in this study;**

Thank you very much for participating in this study.

The aim of this questionnaire study is to provide baseline data. The main aim of this survey is to study the psychological impact of Leishmaniasis among Hubuna's inhabitants at Najran area. The results of this study will ultimately help to overcome undesired consequences. So, it is important that you answer all the questions.

Please read all instruction carefully and answer each question as best you can. Your participation in this research study is voluntary, so you can quit or refuse to participate.

Thank you for your cooperation.

Researchers

***Questionnaire used in the survey***

**A. Demographic Characteristics**

**I will ask you some questions to gather information about your personal information**

**A1.** How old are you/ your child? __________________ **A2.** What is your nationality? -------------------------

**A3.**What is your marital status (single; married; divorced; widow)------------------------

**A4.** What is your educational level? ______________

**A 5.** For how long are you living here in Hubuna? ----------- years

**A6.** Do you have trees or plants inside, surrounded or nearby your home?-----------

**B. Anxiety level due to the disease:**

Using a scale from 0 to 3, put (√)where it indicates your level of agreement with the following statements:

| **Over the course of the disease how often have you been bothered by the following problems?** | not at all or irrelevant  **(0)** | **Nearly every day**  **(1)** | **More than half the days**  **(2)** | nearly every day  **(3)** |
| --- | --- | --- | --- | --- |
| 1.Feeling nervous, anxious or on edge |  |  |  |  |
| 2.Not being able to stop or control worrying |  |  |  |  |
| 3. Worrying too much about different things |  |  |  |  |
| 4. Trouble relaxing |  |  |  |  |
| 5. Being so restless that it is hard to sit still |  |  |  |  |
| 6. Becoming easily annoyed or irritable |  |  |  |  |

**C. Levelof depression due to the disease**

Using a scale from 0 to 3, Please cirlce the number that indicates your level of agreement with the following statements:

| 1 | I do not feel sad. | 0 ( ) |
| --- | --- | --- |
|  | I feel sad | 1 ( ) |
|  | I am sad all the time and I can't snap out of it. | 2 ( ) |
|  | I am so sad and unhappy that I can't stand it. | 3 ( ) |
| 2 | I am not particularly discouraged about the future | 0 ( ) |
|  | I feel discouraged about the future | 1 ( ) |
|  | I feel I have nothing to look forward to. | 2 ( ) |
|  | I feel the future is hopeless and that things cannot improve | 3 ( ) |
| 3 | I do not feel like a failure. | 0 ( ) |
|  | I feel I have failed more than the average person. | 1 ( ) |
|  | As I look back on my life, all I can see is a lot of failures | 2 ( ) |
|  | I feel I am a complete failure as a person. | 3 ( ) |
| 4 | get as much satisfaction out of things as I used to. | 0 ( ) |
|  | I don't enjoy things the way I used to. | 1 ( ) |
|  | I don't get real satisfaction out of anything anymore | 2 ( ) |
|  | I am dissatisfied or bored with everything | 3 ( ) |
| 5 | I don't feel particularly guilty | 0 ( ) |
|  | I feel guilty a good part of the time | 1 ( ) |
|  | I feel quite guilty most of the time. | 2 ( ) |
|  | I feel guilty all of the time | 3 ( ) |
| 6 | I don't feel I am being punished. . | 0 ( ) |
|  | I feel I may be punished | 1 ( ) |
|  | I expect to be punished. | 2 ( ) |
|  | I feel I am being punished | 3 ( ) |
| 7 | I don't feel disappointed in myself. | 0 ( ) |
|  | I am disappointed in myself | 1 ( ) |
|  | I am disgusted with myself. | 2 ( ) |
|  | I hate myself | 3 ( ) |
| 8 | I don't feel I am any worse than anybody else. | 0 ( ) |
|  | I am critical of myself for my weaknesses or mistakes. | 1 ( ) |
|  | I blame myself all the time for my faults. | 2 ( ) |
|  | I blame myself for everything bad that happens | 3 ( ) |
| 9 | I don't have any thoughts of killing myself. | 0 ( ) |
|  | I have thoughts of killing myself, but I would not carry them out. | 1 ( ) |
|  | I would like to kill myself. | 2 ( ) |
|  | I would kill myself if I had the chance | 3 ( ) |
| 10 | I don't cry any more than usual | 0 ( ) |
|  | I cry more now than I used to. | 1 ( ) |
|  | I cry all the time now. | 2 ( ) |
|  | I used to be able to cry, but now I can't cry even though I want to. | 3 ( ) |
| 11 | I am no more irritated by things than I ever was. 1 | 0 ( ) |
|  | I am slightly more irritated now than usual. | 1 ( ) |
|  | I am quite annoyed or irritated a good deal of the time. | 2 ( ) |
|  | I feel irritated all the time. | 3 ( ) |
| 12 | I have not lost interest in other people. | 0 ( ) |
|  | I am less interested in other people than I used to be | 1 ( ) |
|  | I have lost most of my interest in other people. | 2 ( ) |
|  | I have lost all of my interest in other people. | 3 ( ) |
| 13 | I make decisions about as well as I ever could. | 0 ( ) |
|  | I put off making decisions more than I used to. | 1 ( ) |
|  | I have greater difficulty in making decisions more than I used to. | 2 ( ) |
|  | I can't make decisions at all anymore. | 3 ( ) |
| 14 | I don't feel that I look any worse than I used to. | 0 ( ) |
|  | I am worried that I am looking old or unattractive. | 1 ( ) |
|  | I feel there are permanent changes in my appearance that make me look unattractive | 2 ( ) |
|  | I believe that I look ugly. | 3 ( ) |
| 15 | I can work about as well as before. | 0 ( ) |
|  | It takes an extra effort to get started at doing something. | 1 ( ) |
|  | I have to push myself very hard to do anything. | 2 ( ) |
|  | I can't do any work at all. | 3 ( ) |
| 16 | I can sleep as well as usual. | 0 ( ) |
|  | I don't sleep as well as I used to. | 1 ( ) |
|  | I wake up 1-2 hours earlier than usual and find it hard to get back to sleep. | 2 ( ) |
|  | I wake up several hours earlier than I used to and cannot get back to sleep. | 3 ( ) |
| 17 | I don't get more tired than usual. | 0 ( ) |
|  | I get tired more easily than I used to. | 1 ( ) |
|  | I get tired from doing almost anything. | 2 ( ) |
|  | I am too tired to do anything. | 3 ( ) |
| 18 | My appetite is no worse than usual. | 0 ( ) |
|  | My appetite is not as good as it used to be | 1 ( ) |
|  | My appetite is much worse now. | 2 ( ) |
|  | I have no appetite at all anymore | 3 ( ) |
| 19 | I haven't lost much weight, if any, lately. | 0 ( ) |
|  | I have lost more than five pounds. | 1 ( ) |
|  | I have lost more than ten pounds. | 2 ( ) |
|  | I have lost more than fifteen pounds | 3 ( ) |
| 20 | I am no more worried about my health than usual. | 0 ( ) |
|  | I am worried about physical problems like aches, pains, upset stomach, or constipation. | 1 ( ) |
|  | I am very worried about physical problems and it's hard to think of much else. 3 | 2 ( ) |
|  | I am so worried about my physical problems that I cannot think of anything else. | 3 ( ) |
| 21 | I have not noticed any recent change in my interest in sex. | 0 ( ) |
|  | I am less interested in sex than I used to be. | 1 ( ) |
|  | I have almost no interest in sex. | 2 ( ) |
|  | I have lost interest in sex completely. | 3 ( ) |
